# Charge controlled interactions between DNA-modified silica nanoparticles and fluorosurfactants in microfluidic water-in-oil droplets†

**DOI:** 10.1039/d3na00124e

**Published:** 2023-07-05

**Authors:** Sahana Sheshachala, Birgit Huber, Jan Schuetzke, Ralf Mikut, Tim Scharnweber, Carmen M. Domínguez, Hatice Mutlu, Christof M. Niemeyer

**Affiliations:** a Institute for Biological Interfaces (IBG 1), Karlsruhe Institute of Technology (KIT) Hermann-von-Helmholtz-Platz 1 76344 Eggenstein-Leopoldshafen Germany; b Soft Matter Synthesis Laboratory, Karlsruhe Institute of Technology (KIT) Hermann-von-Helmholtz-Platz 1 D-76344 Eggenstein-Leopoldshafen Germany; c Institute for Automation and Applied Informatics (IAI), Karlsruhe Institute of Technology (KIT) Hermann-von-Helmholtz-Platz 1 76344 Eggenstein-Leopoldshafen Germany

## Abstract

Microfluidic droplets are an important tool for studying and mimicking biological systems, *e.g.*, to examine with high throughput the interaction of biomolecular components and the functionality of natural cells, or to develop basic principles for the engineering of artificial cells. Of particular importance is the approach to generate a biomimetic membrane by supramolecular self-assembly of nanoparticle components dissolved in the aqueous phase of the droplets at the inner water/oil interface, which can serve both to mechanically reinforce the droplets and as an interaction surface for cells and other components. While this interfacial assembly driven by electrostatic interaction of surfactants is quite well developed for water/mineral oil (W/MO) systems, no approaches have yet been described to exploit this principle for water/fluorocarbon oil (W/FO) emulsion droplets. Since W/FO systems exhibit not only better compartmentalization but also gas solubility properties, which is particularly crucial for live cell encapsulation and cultivation, we report here the investigation of charged fluorosurfactants for the self-assembly of DNA-modified silica nanoparticles (SiNP-DNA) at the interface of microfluidic W/FO emulsions. To this end, an efficient multicomponent Ugi reaction was used to synthesize the novel fluorosurfactant M4SURF to study the segregation and accumulation of negatively charged SiNP-DNA at the inner interface of microfluidic droplets. Comparative measurements were performed with the negatively charged fluorosurfactant KRYTOX, which can also induce SiNP-DNA segregation in the presence of cations. The segregation dynamics is characterized and preliminary results of cell encapsulation in the SiNP-DNA functionalized droplets are shown.

## Abstract 2

The self-assembly of DNA-modified silica nanoparticles in microfluidic droplets was investigated using a new fluorosurfactant. Such droplets can be used for cell encapsulation.

## Graphical abstract

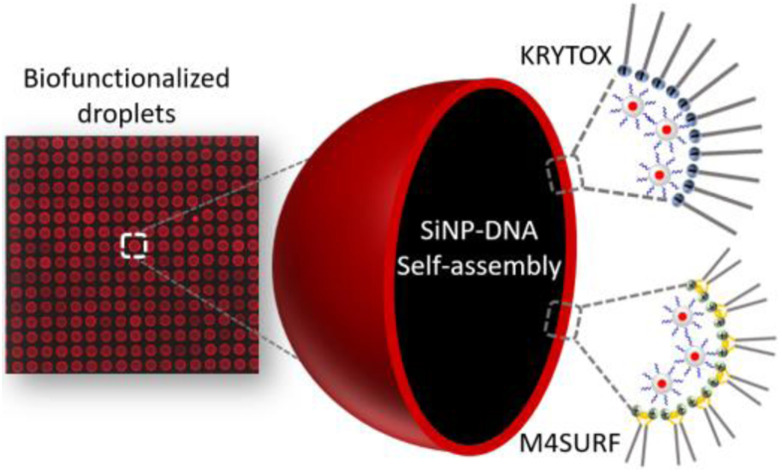

## Introduction

In recent years, microfluidic droplets have been extensively used to mimic natural systems, *e.g.*, to develop synthetic cells and study the interaction of their components.^1–4^ Intensive work is being devoted to bottom-up strategies for the self-assembly of biological components in microfluidic droplets to facilitate specific functions, such as biological protein production, cytoskeleton formation, or microcapsule formation.^5–9^ Customized functionalized droplets can be prepared either by chemically modifying surfactants to induce specific interactions,^10^ or by allowing surfactants to interact with the encapsulated ingredients through non-covalent interactions.^11^ The latter approach has already been used to induce self-assembly of aqueous biological components such as lipid vesicles,^12^ peptides,^13^ proteins^14,15^ and nucleic acids.^7^ For example, DNA-based lattices have been used to functionalize the inner periphery of droplets through electrostatic and hydrophobic interactions. As such, Kurokawa and co-workers^16^ fabricated microfluidically stable liposomes by utilizing the electrostatic interaction between Y-shaped DNA oligomers and cationic lipid surfactants at the droplet interface. By exploiting Watson–Crick base pairing, a polymeric droplet shell was formed from self-assembled DNA that exhibited enhanced elastic modulus and shear modulus.

Along these lines, nanoparticle self-assembly at the liquid–liquid interface of droplets has also been exploited to form mechanically stable droplets that can encapsulate biomolecules and induce cell–ligand interactions.^17,18^ Since DNA-directed self-assembly of proteins is a powerful method to endow arbitrary inorganic surfaces with biomolecular functionality and thus specific interaction potential for living cells,^19–22^ our group recently investigated the self-assembly of DNA-modified silica nanoparticles (SiNP-DNA) at the droplet interface in water–mineral oil (W/MO) emulsions.^23^ Due to the electrostatic interaction between the negatively charged SiNP-DNA and the used cationic lipid 1,2-dioleoyl-3-trimethylammonium propane chloride salt (DOTAP), the formed W/MO droplets exhibited enhanced mechanical stability, which was attributed to the incorporation of silica nanoparticles into hybridized DNA networks. The droplets formed revealed enhanced mechanical stability due to the incorporation of silica nanoparticles into hybridized DNA networks. Specifically, the interaction between negatively charged SiNP-DNA and the cationic lipid 1,2-dioleoyl-3-trimethylammonium propane chloride salt (DOTAP) was used for this purpose. Moreover, the influence of surface charge and biomolecules installed on the SiNP on the kinetics of supramolecular assembly on the inner surface of the DOTAP W/MO droplets was characterized in detail.^24^ To achieve the long-term goal of using microfluidic droplets as containers for biological applications, such as single cell growth and analysis, assembly of SiNPs offers the possibility of creating a more mechanically robust and rigid surface than with pure DNA molecules. Since it was already demonstrated with adherent cells, such as MCF-7, that DNA-conjugated SiNPs form suitable substrates for cell survival and growth,^20^ and furthermore, the use of DNA-modified SiNPs allows modification of the inner surface of microfluidic droplets by DNA-based hybridization strategies to install different biomolecules such as proteins or aptamers on the surface; this approach would create a functional interface in the droplets while avoiding direct contact of the encapsulated cells with the surfactants through the mechanically stable SiNP shell.

**Fig. 1:**
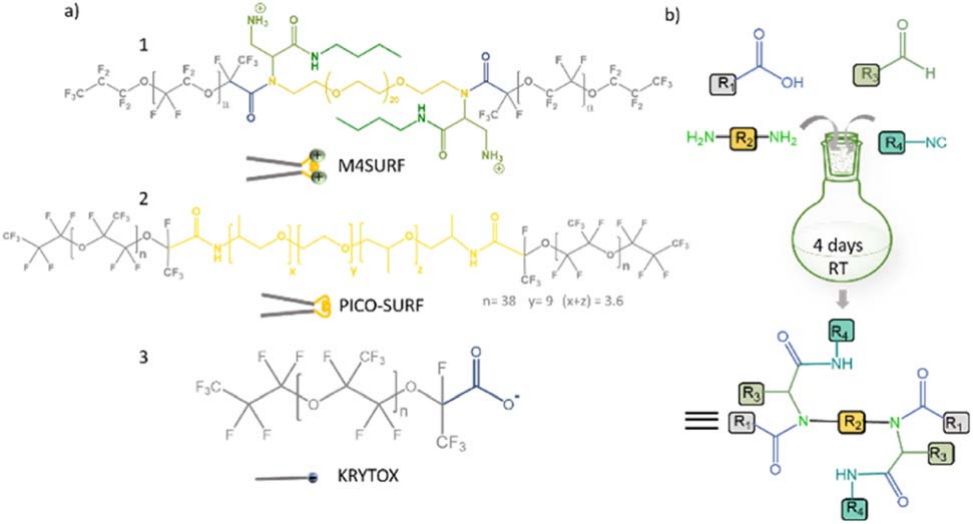
(a) Chemical structures of positively charged M4SURF (1), the neutral PFPE–PEG–PFPE type fluorosurfactant PICO-SURF (2) and negatively charged KRYTOX (3). (b) Schematic illustration of the 4-component Ugi reaction to synthesize M4SURF. Details of the synthesis can be found in the Experimental section and in Fig. S1 in the ESI,† which also includes a discussion on the efficiency and sustainability of the multicomponent Ugi reaction.

However, W/MO droplets are not ideal for biological studies, and recent droplet microfluidic approaches focus on water-in-fluorocarbon oil (W/FO) emulsion droplets for the majority of applications in synthetic biology. Compared to W/MO emulsions, W/FO emulsions not only exhibit better compartmentalization properties due to the hydrophobic and lipophobic fluorocarbon phase,^25^ but also show good solubility for gases, which is crucial for live cell encapsulation and cultivation.^26^ Therefore, it is of great importance to investigate strategies for self-assembly of particles at the droplet interface for W/FO emulsion systems in order to develop opportunities for biomolecular functionalization of the internal droplet interface. Since, to our knowledge, the self-assembly of nanoparticle systems in W/FO emulsions has not yet been investigated, we report here the new fluorosurfactant M4SURF (1 in Fig. 1) that contains positively charged amino groups to enable the electrostatically driven assembly of SiNP-DNA into a thin layer at the internal interface of W/FO droplets.

The novel fluorosurfactant M4SURF, like the vast majority of commercially available fluorosurfactants, contains perfluoropolyether–polyethylene glycol (PFPE–PEG) structures,^27^ such as the widely used fluorosurfactant PICO-SURF (2 in Fig. 1). PICO-SURF and other similar reagents form an inert interface at the aqueous droplet boundary due to the hydrophilic PEG group, but it is uncharged and therefore not suitable for the enrichment and assembly of nanoparticles and other components by electrostatic interactions. The only available charged fluorosurfactant is KRYTOX (3 in Fig. 1) bearing the PFPE tail and a carboxylic head group, thus leading to a negatively charged droplet interface when W/FO emulsions are formed. Indeed, KRYTOX has been used to induce electrostatic interactions with lipid vesicles and polymer compounds at the droplet interface, and the generated droplets were applied for production of hollow, ultrathin microcapsules and solid microparticles,^28^ giant unilamellar vesicles^29^ or as mimics of artificial cells.^30^ However, since the interfacial self-assembly of nanoparticle systems in W/FO emulsions has not yet been explored, we wanted to investigate the suitability of the new positively charged fluorosurfactant M4SURF for this process. For comparison, we also examined whether electrostatic interactions between the SINP-DNA and KRYTOX-stabilized W/FO microdroplets could be achieved by charge reversal using dissolved cations. We demonstrate and characterize the segregation and accumulation of SiNP-DNA dispersed in the aqueous phase at the charged inner interface of M4SURF and KRYTOX-stabilized microfluidic droplets, and show preliminary results that cells can be encapsulated in the functionalized droplets.

## Results and discussion

To develop new chemical synthetic approaches for fluorosurfactant availability, we aimed to explore for the first time whether a green and highly efficient multicomponent reaction (MCR) strategy can be used for the synthesis of the novel fluorosurfactant M4SURF. MCRs are one-pot reactions in which three or more reactants form a single product with minimal effort and high efficiency and which have become an indispensable tool for the design of complex molecules and diverse drug libraries.^31^ One of the most commonly used MCRs is the 4-component Ugi reaction of carboxylic acid, amine, carbonyl, and isocyanide derivatives, which forms a final product with a stable bis-amide bond.^32^ Accordingly, as shown in Fig. 1b, a simple mixture of KRYTOX (as the carboxylic acid component, R_1_ in Fig. 1b) and a PEG-diamine (R_2_) with an *N*-Boc-aldehyde (R_3_) and an isocyanobutane (R_4_) component yielded the novel fluorosurfactant M4SURF. The successful conversion of KRYTOX with the MCR strategy to yield M4SURF was confirmed using IR and ^19^F NMR spectroscopy as well as gel permeation chromatography (Fig. S1, ESI†). IR data allowed us to conclude that M4SURF is positively charged, in line with previous works^33–37^ (see Fig. S1† for a detailed explanation). While the spectroscopic and chromatographic analyses were in agreement with expectations (Fig. S1†), the ^1^H NMR spectra did not provide clear information due to solubility problems in the common solvents (C_6_D_6_, C_6_F_6_).

The synthesized tri-block fluorosurfactant M4SURF contains two positively charged amino groups and a central PEG moiety in the hydrophilic domain linked to two PFPE tails in the fluorophilic domain, and thus has a similar structure to commonly available PFPE–PEG–PFPE type droplet stabilizing surfactants, such as PICO-SURF. Overall, the above data clearly showed that MCR is a promising strategy for the synthesis of fluorosurfactants. The MCR-Ugi strategy used here offers advantages over conventional synthetic routes for fluorosurfactants, which can be limited by tedious multistep reactions^38^ or lower structural variability of orthogonal coupling methods.^39,40^ The MCR-Ugi strategy presented here should greatly facilitate, for example, the synthesis of libraries of functional fluorosurfactants with different interfacial properties by changing the structure of the hydrophilic headgroup and/or inducing specific interactions within the droplets by attaching (bio)functional interaction groups.

To investigate the possible recruitment of silica nanoparticles at the inner surface of W/FO microfluidic droplets, we employed SiNP modified with DNA (SiNP-DNA) that were synthesized according to a previously described reverse micelle method^41^ (Fig. S2†) using the aminoalkyl-modified oligonucleotide aP1 (for the sequence, see Table S1†). The SiNP were encoded with the fluorescent dye Abberior STAR 635 to allow for microscopic visualization. The synthesized SiNP-DNA contained about 132 ± 16 copies of ssDNA oligomers per particle on their surface, as determined by the supernatant depletion method, and showed zeta potential values around −42.5 mV, confirming the colloidal stability of the dispersion.

**Fig. 2:**
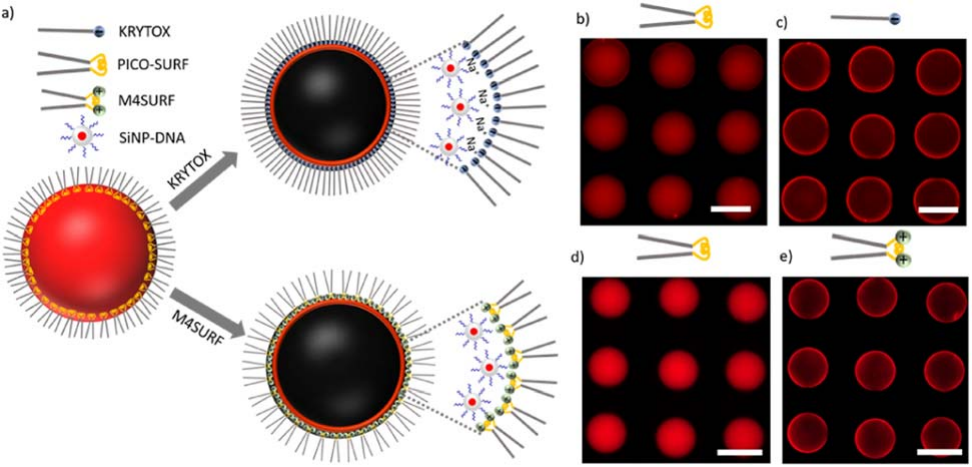
(a) Schematics of the charge-induced segregation of colloidal silica-DNA nanoparticles (SiNP-DNA) dispersed in PBS buffer containing 10 mM NaCl; (b–e) fluorescence images of W/FO droplets in the presence of three different surfactants, PICO-SURF (b and d), KRYTOX (c) and M4SURF (e). Note that the droplets in (c) and (e) were formed from the droplets in (b) and (d) after *in situ* addition of KRYTOX and M4SURF, respectively. Scale bars are 100 μm.

The novel fluorosurfactant M4SURF as well as KRYTOX were then tested for their ability to interact with SiNP-DNA at the droplet interface. Since both SiNP-DNA and KRYTOX are negatively charged, the assembly of SiNP-DNA in the inner droplet interface was not expected in the presence of KRYTOX as a surfactant. However, inspired by initial work to overcome repulsion between negatively charged vesicles by using Mg^2+^,^29^ we used SiNP-DNA in the presence of cations (Na^+^ and K^+^) to mediate the electrostatic association between the two negatively charged components, SiNP-DNA and KRYTOX, respectively. Fig. 2a shows the schematic of the charge-induced assembly of SiNP-DNA and the fluorosurfactants. Microfluidic droplets were produced from an aqueous phase containing SiNP-DNA (4 mg mL^−1^) in PBS buffer (23 mM KH_2_PO_4_, 77 mM K_2_HPO_4_, pH 7.6) that was supplemented with 10 mM NaCl. The concentration of the particles was chosen based on previous work, as this achieves sufficient segregation dynamics and the formation of a dense shell of assembled particles at the interface.^24^ The selection of the PBS buffer containing 177 mM K^+^ cations was also based on this study, as the buffer ensures sufficient colloidal stability and biocompatibility. The increase in cation concentration by 10 mM Na^+^ was based on previous studies on the interaction of these cations with DNA. For example, molecular dynamics simulations revealed that Na^+^ ions preferentially interact with the phosphate groups of DNA molecules, while K^+^ ions bind mainly to the electronegative sites of DNA bases in the major and minor grooves.^42^ Moreover, Na^+^ ions condense around DNA to a greater extent and show a much stronger effect in compacting DNA chains into condensed superstructures compared to K^+^.^43^

The fluorinated oil HFE 7500, supplemented with the non-ionic surfactant PICO-SURF (1 wt%), was used as the oil phase, and either M4SURF (10 wt%) or KRYTOX (10 wt%) in HFE 7500 was used to replace the PICO-SURF and thereby to induce self-assembly of SiNP-DNA at the droplet interface. Note that this exchange step was necessary to visualize and track the initial segregation kinetic parameters, which would not have been possible experimentally had we used directly charged surfactants. Although the exact distribution of the different surfactants PICO-SURF, KRYTOX and M4SURF cannot be determined experimentally, based on previous results^29^ and given the low viscosity of the PICO-SURF in HFE 7500 solution (1.24 cP, according to manufacturer's data, Sphere Fluidics), it can be assumed that most of the PICO-SURF is washed out and exchanged for M4SURF or KRYTOX at the water–oil interface. Since KRYTOX and M4SURF have significantly different molecular weights (about 7500 and 16 500 g mol^−1^, respectively) and since the concentration of surface-active molecules have an impact on the dynamics of the segregation process, detailed studies with precisely assessed molarity would be required to allow precise quantitative conclusions. However, for reasons of practicality, comparable weight percentages typically described in the literature were initially used here to provide a qualitative indication of the effectiveness of the two strategies. The droplets were produced using a flow-focusing microfluidic chip made of PDMS and stored in on-chip storage (OCS) chambers, which enabled spatial separation of the droplets inside designed wells (Fig. S3a and S3b†), as previously established in our laboratory.^24,44,45^ Furthermore, the PDMS microchannels were rendered fluorophilic by coating them with perfluorooctyltrichlorosilane before use (see the Experimental section). W/FO droplets formed with the three different surfactants used in this work could be collected and stored in a vial for several days at either RT or 37 °C, indicating comparably high stability (Fig. S3c†).

The results of initial assembly experiments are shown in Fig. 2b. In the presence of PICO-SURF (Fig. 2b and d) no apparent self-assembly occurred, as expected from the non-ionic nature of this surfactant. In contrast, when KRYTOX and M4SURF were applied as surfactants (Fig. 2c and e, respectively), the fluorescence gradually shifted towards the inner surface of the droplets, thereby indicating attractive interactions between SiNP-DNA and the fluorosurfactants. The result obtained for the negatively charged KRYTOX (Fig. 2c) confirmed the hypothesis that cations can indeed act as bridging elements, mediating the formation of supramolecular assemblies between the otherwise repulsive, negatively charged surfaces. The necessity of cations for the formation of the supramolecular SiNP-DNA layer was also confirmed by control experiments in which the SiNP-DNA was dispersed in ultrapure water and therefore remained uniformly distributed inside the droplet (Fig. S4a†). In contrast, the novel cationic fluorosurfactant M4SURF showed the expected electrostatic accumulation at the droplet interface even when the SiNP-DNA was dispersed in ultrapure water (Fig. S4b†).

To further elucidate the nature of the charge-mediated assembly of SiNP-DNA with KRYTOX and M4SURF (SiNP-DNA/KRYTOX and SiNP-DNA/M4SURF systems, respectively), the segregation kinetics of fluorescent SiNP-DNA inside the droplets were further investigated by varying the concentrations of fluorosurfactants (*C*_KRYTOX_ and *C*_M4SURF,_ respectively) and NaCl in PBS buffer (*C*_NaCl_). To examine the segregation dynamics, in a first set of experiments, microfluidic droplets (around 100 μm in diameter) were initially produced with SiNP-DNA in PBS as the aqueous phase and HFE 7500/PICO-SURF as the continuous phase. After capturing the droplets inside the wells of the OCS chamber, the HFE 7500/PICO-SURF oil phase was replaced with HFE 7500 oil containing variable concentrations of KRYTOX or M4SURF and passed through the chip at a constant flow rate of 2 μL min^−1^. This allowed KRYTOX or M4SURF to replace the PICO-SURF surfactant in the droplets over time.

The droplets collected in the OCS wells allowed the measurement of the time-dependent fluorescence changes of SiNP-DNA inside the droplets by fluorescence microscopy (Fig. S5†). With the microfluidic chip, up to nine droplets could be monitored in a single image, enabling observation with relatively high throughput. Fluorescent images were taken every 2 s for 10 min. The obtained image series of the droplets were analyzed by means of an automated MATLAB pipeline, previously developed in our laboratory^24^ (Fig. S5†), that allows quantification of the kinetic segregation parameters (*t*_1/2_ and *k*_s_). Since the image series had nine droplets in each image, the automated analysis was adopted here to detect and analyze multiple droplets within a single input image. The model *f*(*t*) = *a* − *b* e^−*c*(*t*−*d*)^ was used to fit the asymptotic assembly curves. The quantifying parameter ‘*t*_1/2_’ describes the time (in minutes) at which half of the segregating particles have reached the interface and was calculated using the fit parameters of the asymptotic curve. The first derivative of the fit yielded ‘*k*_s_’ – the segregation rate (min^−1^) of SiNP-DNA. Note that a decrease in *t*_1/2_ is accompanied by an increase in the *k*_s_ value, indicating a faster segregation process. For simplicity, *t*_1/2_ values are used in the following to compare different data sets.

**Fig. 3:**
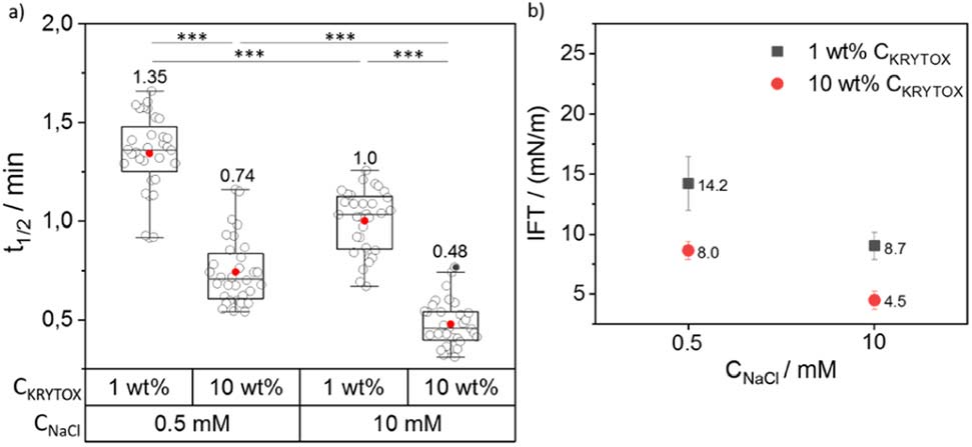
(a) Grouped box plot of *t*_1/2_ values obtained for the SiNP-DNA interface assembly with a varying weight percentage (1 wt% and 10 wt%) of KRYTOX in HFE 7500 oil against concentrations of 0.5 and 10 mM NaCl. The box plot shows the median and 25–75th percentiles. Individual data points, mean and outliers are represented by hollow, red and black dots, respectively. 33 droplets were analyzed from 5 independent experiments for each condition. The corresponding mean values are also indicated above each box plot. (b) Interfacial tension (IFT) values for SiNP-DNA/KRYTOX systems obtained at the variable concentrations of KRYTOX and NaCl used in this study, as analyzed using 10–15 pendant droplets. A two-way ANOVA was performed using SPSS software to analyze the effect of *C*_NaCl_ and *C*_KRYTOX_ on the *t*_1/2_. The test revealed that there was no statistically significant interaction between the effects of *C*_NaCl_ and *C*_KRYTOX_ (*p* = 0.181). Simple main effects analysis showed significant effects on the *t*_1/2_ that are indicated by asterisks (***p < 0.001). Outliers were determined according to Tukey's formula [Q1 − 1.5 IQR; Q3 + 1.5 IQR].

To investigate the influence of the concentration of NaCl and KRYTOX or M4SURF on the segregation, concentrations of either 1 wt% or 10 wt% KRYTOX or M4SURF in HFE 7500 oil as well as PBS containing NaCl concentrations of either 0.5 and 10 mM were used. For the choice of salt concentrations, we were guided by the fact that PBS (phosphate-buffered saline, with a composition of 23 mM KH_2_PO4, 77 mM K_2_HPO4, and varying amounts of NaCl) is commonly used as a buffer for biological studies. Therefore, we decided to use this buffer composition here as well, so that neither the pH nor the ionic strength (total 177 mM K^+^ ions) was changed, while testing a low and a relatively high Na^+^ concentration, *i.e.*, 0.5 and 10 mM Na^+^. The *t*_1/2_ and *k*_s_ values were calculated for each set of experiments. The data obtained for *t*_1/2_ values were used for comparing the effect of the *C*_KRYTOX/M4SURF_ and *C*_NaCl_. Fig. 3 shows the grouped box plot of *t*_1/2_ values obtained for SiNP-DNA in dependency of *C*_NaCl_ and *C*_KRYTOX_ (for the respective *k*_s_ values, see Fig. S6†).

It is evident from Fig. 3a that the *t*_1/2_ value decreases with increasing *C*_KRYTOX_ from 1 wt% to 10 wt% from 1.35 min to 0.74 min at 0.5 mM *C*_NaCl_. A similar trend was observed at 10 mM *C*_NaCl_ (1 min to 0.48 min). The same trend was also reflected by the respective slope values (*k*_s_), where an increase in *C*_KRYTOX_ resulted in higher *k*_s_ values (Fig. S6†). Thus, by increasing the concentration of KRYTOX from 1 wt% to 10 wt%, the interfacial coverage with the negatively charged surfactant molecules increased, resulting in faster assembly of SiNP-DNA at the interface and thus lower *t*_1/2_ and higher *k*_s_ values. Because of the constant flow rate (2 μL min^−1^) employed for the flow of incoming KRYTOX, an increase in the KRYTOX concentration from 1 wt% to 10 wt% increases the coverage of KRYTOX at the water–oil interface, leading to reduced *t*_1/2_ values. It is also evident from Fig. 3a that an increase in Na^+^ concentration (in PBS) resulted in decreased *t*_1/2_ values for both KRYTOX concentrations investigated (1 wt% and 10 wt%), thereby indicating a faster assembly of SiNP-DNA at the interface. This finding is in agreement with previous work, indicating that bivalent Mg^2+^ cations can bridge two negatively charged moieties to enable formation of biomolecular assemblies at the droplet interface.^12^ Note that this change in *t*_1/2_ occurs with a 20-fold increase in Na^+^ concentration despite the constant, quite high concentration of K^+^ ions (177 mM) in the PBS buffer. Therefore, the results obtained here confirm the assumption made above, based on previous studies,^42,43^ that Na^+^ ions cause a much stronger shielding of the negatively charged phosphate groups of DNA than K^+^ ions also in this segregation process.

The interfacial interaction of the SiNP-DNA/KRYTOX system was also analyzed using pendant drop tensiometry to measure the interfacial tension (IFT) between SiNP-DNA and KRYTOX in aqueous and fluorocarbon phases, respectively (for the detailed measurement setup, see Fig. S7†). IFT is a widely used parameter to characterize the interfacial activity of liquid–liquid interfaces, particularly for emulsions,^46,47^ and it can also be used to evaluate the behavior of nanoparticles and surfactants at the interface of W/O droplets.^17^ Complementary interactions between nanoparticles and surfactants lead to an increase in the surface coverage of the interacting components, in this case SiNP-DNA with KRYTOX, at the water–oil interface, which should decrease the IFT of the system. The higher the surface coverage of interacting surfactants and nanoparticles at the interface, the lower the IFT of the system.^48^ The surface coverage at the water–oil interface depends on the concentration of nanoparticles in the aqueous phase and surfactants in the oil phase, and in turn affects the IFT of the system.^49^ Since the driving force between the SiNP DNA and the fluorine-containing surfactants is electrostatic interactions, the ionic strength of the aqueous phase also has a significant effect on the interfacial arrangement and thus can lead to changes in the interfacial tension of the system.

The IFT data obtained for the SiNP-DNA/KRYTOX system (Fig. 3b) indicated IFT values of 14 mN m^−1^ (1 wt% *C*_KRYTOX_) or 8 mN m^−1^ (10 wt% *C*_KRYTOX_) for the SiNP-DNA/KRYTOX system at 0.5 mM *C*_NaCl_. While the trend was similar for 10 mM *C*_NaCl_, the IFT value decreased with an increase in the surfactant concentration from 8.7 mN m^−1^ (1 wt% *C*_KRYTOX_) to 4.5 mN m^−1^ (10 wt% *C*_KRYTOX_). This indicated that the interfacial surface coverage of the droplet is higher when the KRYTOX concentration increases, leading to the observed faster assembly (lower *t*_1/2_ values) of SiNP-DNA at the water–oil interface. This observation is in agreement with a study on the interaction of transition metal nanoparticles with polymeric surfactants,^50^ indicating an increase in interfacial assembly with increasing surfactant concentrations.

**Fig. 4:**
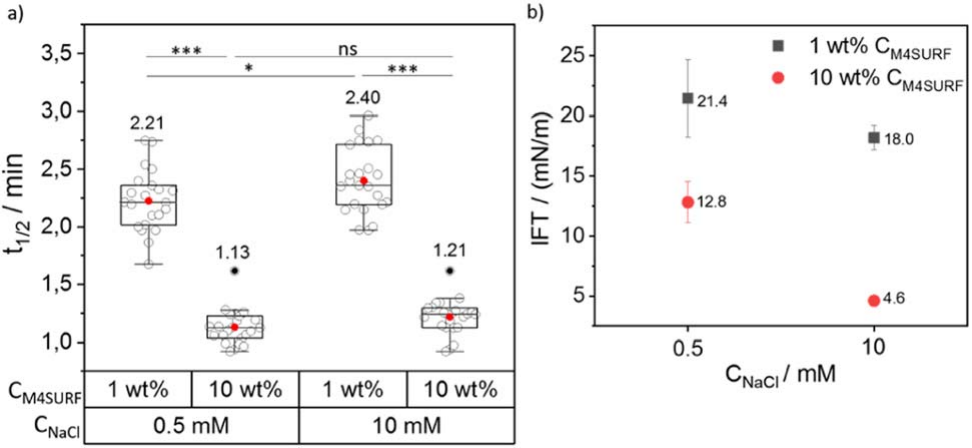
(a) Grouped box plot of *t*_1/2_ values obtained for SiNP-DNA interface assembly with a varying weight percentage (1 wt% and 10 wt%) of M4SURF (a) in HFE 7500 oil, against concentrations of 0.5 and 10 mM NaCl. The box plot shows the median and 25–75th percentiles. Individual data points, mean and outliers are represented by hollow, red and black dots, respectively. 23 droplets were analyzed from 4 independent experiments for each condition. The corresponding mean values are also indicated above each box plot. (b) Interfacial tension (IFT) values for SiNP-DNA/M4SURF systems obtained at the variable concentrations of M4SURF and NaCl used in this study, as analyzed using 10–15 pendant droplets. A two-way ANOVA was performed using SPSS software to analyze the effect of *C*_NaCl_ and *C*_M4SURF_ on the *t*_1/2_. The test revealed that there was no statistically significant interaction between the effects of *C*_NaCl_ and *C*_M4SURF_ (*p* = 0.356). Simple main effects analysis showed significant effects on the *t*_1/2_ that are indicated by asterisks (*p < 0.05; ***p < 0.001). Outliers were determined according to Tukey's formula [Q1 – 1.5 IQR; Q3 + 1.5 IQR].

We then investigated the influence of positively charged M4SURF on the segregation rate of SiNP-DNA. Fig. 4a shows the grouped box plot of *t*_1/2_ values for the SiNP-DNA/M4SURF system. Although, due to the differences in molecular weight and molarity mentioned above, the results obtained for M4SURF cannot be quantitatively compared with those obtained for KRYTOX, the results show that the positively charged M4SURF affected the segregation rate of SiNP DNA in a similar manner to the KRYTOX/NaCl system. Again, higher M4SURF concentrations led to lower *t*_1/2_ values, and this trend was also reflected in the *k*_s_ values (Fig. S6b†). The IFT data obtained for the SiNP-DNA/M4SURF system also followed a similar trend to those measured for the KRYTOX system (Fig. 4b). In particular, a decrease in IFT values for the M4SURF system was observed with an increase in surfactant concentration at both NaCl concentrations (21.4 mN m^−1^ (1 wt% *C*_M4SURF_) and 12.8 mN m^−1^ (10 wt% *C*_M4SURF_) at 0.5 mM *C*_NaCl_, and 18 mN m^−1^ (1 wt% *C*_M4SURF_) and 4.6 mN m^−1^ (10 wt% *C*_M4SURF_) at 10 mM *C*_NaCl_). As in the case of KRYTOX, this indicated that the interfacial coverage of the droplet is higher when the M4SURF concentration increases, leading to the observed faster assembly (lower *t*_1/2_ values) of SiNP-DNA at the water–oil interface.

Fig. 4 also shows that increasing the Na^+^ concentration from 0.5 mM to 10 mM in the aqueous buffer phase (containing SiNP DNA) for a given M4SURF concentration resulted in minimal increase (1 wt%) or no significant (10 wt%) change in *t*_1/2_ values, unlike the KRYTOX case. This is consistent with the expectations that with increasing NaCl concentration, the shielding of the SiNP-DNA charge (responsible for the electrostatic interaction with oppositely charged M4SURF molecules) would also increase, slowing down the assembly of SiNP-DNA at the interface. However, the SiNP-DNA/M4SURF system (in both cases of 1 wt% and 10 wt% M4SURF) showed a reduction in IFT upon increase of *C*_NaCl_ (21.4 mN m^−1^ to 18 mN m^−1^ at 1 wt% *C*_M4SURF_ or 12.8 mN m^−1^ to 4.6 mN m^−1^ at 10 wt% *C*_M4SURF_, Fig. 4b), a similar trend to that observed with the KRYTOX system. Indeed, this observation is consistent with the literature showing that higher surface coverage at the liquid–liquid interface is achieved with both positively and negatively charged surfactants when the concentration of NaCl in the aqueous phase is increased.^51–53^ Thus, the lower IFT values result from the higher surface coverage of the interface with M4SURF at increased NaCl concentration and two opposing effects occur. On the one hand, the KRYTOX experiments show that increased NaCl concentration leads to increased charge shielding of negatively charged surfactant and SiNP-DNA and thus to altered electrostatic interaction between the particles and surface. Although the SiNP-DNA shielding inhibits the interaction with the positively charged M4SURF, increasing NaCl concentration also increases the number of charged M4SURF molecules at the interface, which in turn leads to a stronger interaction between the particles and surface. These opposing mechanisms provide a plausible explanation for why no relevant changes in NaCl-dependent segregation kinetics were observed for the SiNP-DNA/M4SURF system.

Overall, the comparison of the KRYTOX and M4SURF systems showed that for the negatively charged KRYTOX, a 20-fold increase in Na^+^ concentration leads to an approximately 30% increase in assembly velocity, while for the positively charged M4SURF, as expected, this is barely affected. Furthermore, the comparison showed that a 10-fold increase in KRYTOX or M4SURF concentration almost doubles the assembly rate, which allows interesting conclusions to be drawn about the underlying mechanisms. Therefore, further studies with systematically changed K^+^ and Na^+^ concentration could be used, for example, for quantitative comparison of the shielding efficiency by the two alkali cations.

Having gained a basic understanding of the kinetics of electrostatic interactions between DNA-SiNP and the fluorosurfactants KRYTOX and M4SURF in the microfluidic droplets, we wanted to investigate whether such particle-stabilized droplets could also be used to encapsulate adherent cells and whether the cells would then interact with the SiNP-DNA droplet interface. Adherent MCF-7 breast cancer cells expressing the Epidermal Growth Factor (EGF) receptor genetically fused with an enhanced green fluorescent protein (eGFP-EGFR), denoted as MCF-7_eGFP_, were used for preliminary experiments. Previous studies had demonstrated that these well characterized cells enable the convenient fluorescence microscopy observation of the cellular membrane.^54^ The concentration of MCF-7_eGFP_ cells was adjusted statistically (following the Poisson distribution, see Fig. S8†) to obtain droplets with one or two cells.^55^

**Fig. 5:**
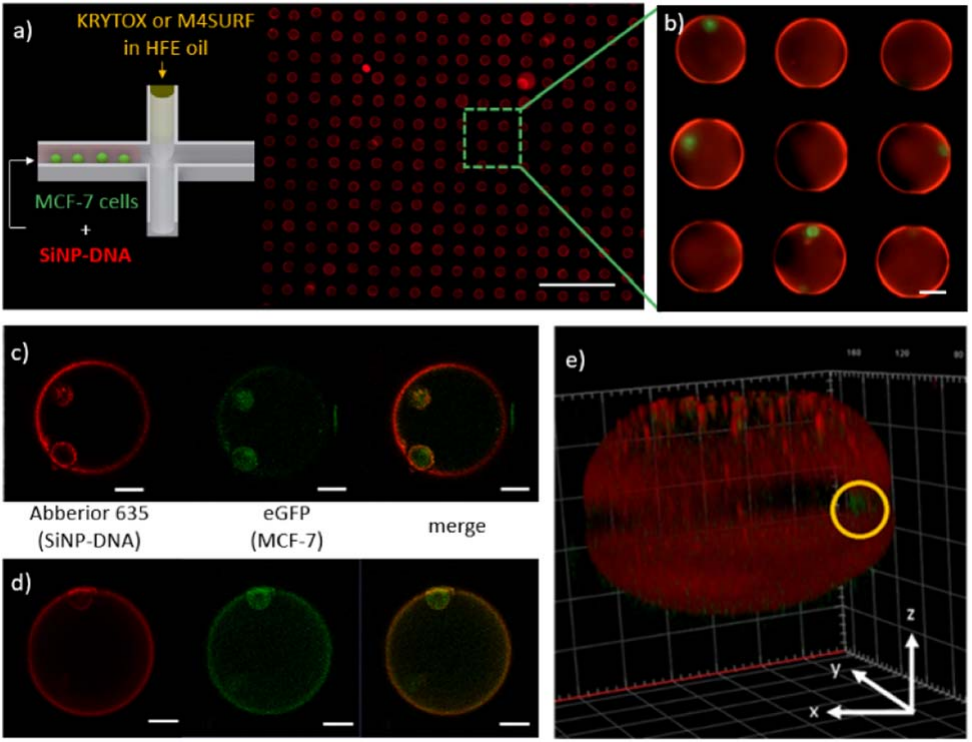
(a) Schematic representation of the encapsulation of MCF-7_eGFP_ cells with SiNP-DNA in cell culture medium (left) and a representative fluorescence image of the microfluidic chip containing an array of droplets inside the on-chip storage wells (right). The scale bar is 500 μm. (b) Enlarged image of microfluidic droplets containing MCF-7 cells and self-assembled SiNP-DNA. Scale bar is 50 μm. (c and d) 2D confocal images of the MCF-7_eGFP_ cells (green) inside microfluidic droplets obtained with the SiNP-DNA/KRYTOX (c) and SiNP/M4SURF (d) systems. The shell-like structures (red) contain the MCF-7_eGFP_ cells (green) that adhered to the SiNP-DNA-decorated interface. Scale bars are 25 μm. (e) 3D reconstruction image of a representative microfluidic droplet containing an encapsulated MCF-7_eGFP_ cell (green, marked by a yellow circle) and the self-assembled SiNP-DNA layer.

Fig. 5a shows an array of droplets which reveal shell-like structures that formed spontaneously due to the interaction between the SiNP-DNA (shown in red) and the KRYTOX or M4SURF, respectively. Encapsulated MCF-7_eGFP_ cells (green) are clearly visible in the enlarged image of the self-assembled SiNP-DNA droplets (Fig. 5b). Confocal fluorescence microscopy images of MCF-7_eGFP_ cells (green) inside the microfluidic SiNP-DNA/KRYTOX (Fig. 5c) and SiNP-DNA/M4SURF (Fig. 5d) droplets taken 2 h after encapsulation show cell attachment to the inner surface of the droplet. Moreover, 3D reconstruction of a Z-stack image of the droplet (Fig. 5e) clearly shows that the MCF-7_eGFP_ cells directly adhere to the particle shell in the equatorial plane of the droplet instead of gravitationally sedimenting to the droplet's bottom. This indicates a high affinity of the cells for the thin SiNP-DNA layer at the droplet boundary and is consistent with previous studies on the adhesion of MCF-7_eGFP_ cells to SiNP-DNA layers on planar support substrates. Indeed, it is already well documented that DNA-modified SiNP are highly attractive for MCF-7_eGFP_ cell culture applications.^20–22^

We note that the results shown above provide only a first positive indication of potential applications of the strategy reported here for the preparation of particle-stabilized W/FO microdroplets. Further detailed studies need to follow to systematically investigate, for example, the cell viability, growth and division ability in different systems, such as SiNP-DNA/M4SURF or SiNP-DNA/KRYTOX. However, the studies on long-term cultivation of the cells in the W/FO emulsion droplets remain a challenge that requires further technical optimization, especially for the storage of the droplets in or outside the OCS chambers at optimal temperatures (37 °C) and O_2_/CO_2_ atmospheric conditions for cell growth, for example to minimize interfering evaporation of the perfluorinated phases. In this work, the emulsions were also transferred to vials and broken with perfluoro-octanol. However, the perfluorooctanol treatment did not prove ideal for cell survival, so alternative methods are currently being sought. It seems reasonable that these technical problems are solvable and that the SiNP-DNA droplets described here could be used as hollow spherical 3D microreactors in which a self-assembled SiNP-DNA layer serves as a tailored cell culture substrate for adherent cells.

## Experimental

### Materials

Tetraethyl orthosilicate (99%, TEOS, Merck), (3-mercaptopropyl)trimethoxysilane (95%, MPTMS, Merck), N1-(3-trimethoxysilylpropyl)diethylenetriamine (DETAPTMS, Merck), (3-aminopropyl)triethoxysilane (99%, APTES, Merck), ammonium hydroxide (25%, Merck), Abberior STAR 635 NHS ester (Abberior), cyclohexane (Merck), 1-hexanol (Merck), triton-X-100 (Merck), dichloromethane (Merck), hexafluoroisopropanol (Merck), poly(ethylene glycol) bis(3-aminopropyl) terminated (Merck, Mn ∼1500), 1-isocyanobutane (Merck), *tert*-butyl *N*-(2-oxidanylideneethyl)carbamate (*i.e.*, *N*-Boc-aldehyde, Merck), glutaraldehyde (50% in water, Merck), (1*H*,1*H*,2*H*,2*H*-perfluorooctyl)-trichlorosilane (Merck), trifluoroacetic acid (Merck), PICO-SURF (Dolomite microfluidics), KRYTOX FSH, HFE 7500 fluorinated oil (Costenoble), sodium chloride (NaCl, VWR chemicals), fluorescamine (98%, Merck), tris(2-carboxyethyl)phosphine hydrochloride solution (TCEP, 0.5 M, Merck), sodium dihydrogen phosphate (Roth), disodium hydrogen phosphate (Merck), PDMS base polymer and crosslinker (Dow Corning), MCF-7_eGFP_ (MPI, Dortmund), penicillin–streptomycin (Thermo Fischer Scientific), EMEM cell culture medium (ATCC), DPBS (Gibco), and oligonucleotides (Merck).

### Synthesis of DNA-modified SiNP (SiNP-DNA)

SiNP modified with an amino-alkyl modified oligonucleotide (aP1, for the sequence, see Table S1†) was synthesized according to a previously established protocol.^41^ The particles were stored at 4 °C in 1× Tris buffer.

### Synthesis of M4SURF fluorosurfactant

M4SURF is a positively charged fluorosurfactant synthesized in-house to interact electrostatically with negatively charged SiNP-DNA. It was synthesized by a multicomponent Ugi reaction. Typically, KRYTOX FSH (500 mg, 68.5 μmol, 2.00 equiv.) was dissolved in HFE 7500 (1 mL) in a roundbottom flask and stirred under an inert atmosphere. In a second flask PEG diamine (51 mg, 34 μmol, 1.00 equiv.) and *N*-Boc-2-aminoacetaldehyde (10.9 mg, 68.5 μmol, 2.0 equiv.) were dissolved in 1 mL dry dichloromethane under an inert atmosphere. This solution was transferred to the KRYTOX/HFE solution and 1-isocyanobutane (5.7 mg, 7.2 μL, 68.5 μmol, 2.0 equiv.) was added. The reaction was vigorously stirred for four days at room temperature. The product was precipitated in diethyl ether twice. The diethyl ether was decanted, the residue was dissolved in HFE 7500 and evaporated to give 445 mg of transparent viscous product (yield: 83%). The transparent, viscous product (445 mg, 28 μmol, 1.00 equiv.) was dissolved in 2 mL HFE. Under a N_2_-flow 2,2,2-trifluoroacetic acid (4.8 g, 3.2 mL, 41.8 mmol, 1500 equiv.) was added *via* a syringe pump over 2 h at 0 °C. The obtained product was purified by dialysis in water (cellulose membrane of 2000 Da/3 days) and freeze dried to obtain positively charged M4SURF (yield ∼86%). The formation of the product was analyzed by gel permeation chromatography. The purity of the product was analyzed using infrared spectroscopy (Fig. S1†).

### Physicochemical characterization of SiNP-DNA

The hydrodynamic size and zeta potential of SiNP were analyzed at room temperature using a Malvern Zetasizer, Nano ZSP, equipped with a standard 633 nm laser. Transmission electron microscopy (TEM, EM910, Carl Zeiss) was used to measure the size of the nanoparticles.

### Quantification of functional groups

The quantification of amine and thiol groups on DNA modified SiNP was achieved by the fluorescamine assay and Ellman's assay, respectively, using the previously described protocol.^41^ The conjugated oligonucleotides were quantified using the previously described supernatant depletion method by measuring absorbance at 260 nm and hybridization assay using fluorescent complementary oligonucleotides (Cy3-cP1) measured at 570 nm with an excitation wavelength of 510 nm using a TAKE3 plate in a microplate reader.

### Infrared spectroscopy (IR)

FT-IR spectra were recorded using an attenuated total reflectance infrared spectroscopy (ATR-IR, Smart iTR) unit on a Bruker VERTEX 80 V FT-IR spectrometer in the range of 600–4000 cm^−1^ at ambient temperature.

### Gel permeation chromatography (GPC)

GPC measurements were performed in hexafluoroisopropanol (HFIP) containing 0.1 wt% potassium trifluoroacetate using a Tosoh EcoSEC HLC-8320 SEC system. The solvent flow was 0.40 mL min^−1^ at 30 °C and the concentration of the samples was 1 mg mL^−1^. The analysis was performed on a three-column system: PSS PFG Micro precolumn (3.0 × 0.46 cm, 10 000 Å), PSS PFG Micro (25.0 × 0.46 cm, 1000 Å) and PSS PFG Micro (25.0 × 0.46 cm, 100 Å). Linear poly(methyl methacrylate) standards (Polymer Standard Service, Mp: 102–981 kDa) were used to calibrate the system.

### Interfacial tension (IFT) measurements

Interfacial tension measurements were performed using the pendant drop method in a custom-made setup, kindly provided by colleagues at the Institute of Mechanical Process Engineering and Mechanics (MVM), KIT. Oil droplets of HFE 7500 containing variable concentrations of KRYTOX/M4SURF were generated manually through a 1 mL syringe with a 0.8 mm needle inside the aqueous solution containing varying concentrations of SiNP-DNA in PBS (for the detailed setup, see Fig. S7†). The shape of the hanging drop was detected and fitted with the Young–Laplace equation using the droplet shape analysis (DSA) software to derive the interfacial tension (IFT) between the two phases.

### Microfluidic chip fabrication

To prepare PDMS chips, polydimethylsiloxane (PDMS) was mixed with the curing agent in a ratio of 10 : 1 and degassed in a vacuum chamber for 1 h. Subsequently, the PDMS was poured into a PMMA mold and cured at 70 °C for 1.5 h. The cast chip was finally bonded to a Cyclo Olefin Polymer (COP) film to seal the chip and was used for droplet generation. PMMA molds were prepared using the micro-milling technique according to a previously described technique.^44^

### Microfluidic generation of water-in-oil (W/O) droplets

For generating W/FO droplets, the PDMS microchannels were coated with (1*H*,1*H*,2*H*,2*H*-perfluorooctyltrichlorosilane) (1% (vol/vol) solution in isopropanol) to make the surface fluorophilic. To this end, the PDMS chips were first activated by oxygen plasma to generate hydrophilic microchannels. The freshly oxidized PDMS chip was dipped in the solution containing fluorosilane for 20 min. The chip was then washed with isopropanol several times and flushed with N_2_ and dried in an oven at 70 °C before use. SiNP-DNA (4 mg mL^−1^) particles were dispersed either in ultrapure water or PBS (containing 0.5, 10 and 50 mM NaCl) and the fluorosurfactant (PICO-SURF, 1 wt%) was dissolved in HFE 7500. Droplets were produced using a PDMS chip with a flow-focusing junction. Initially, the flow rates were 0.5 μL min^−1^ and 23 μL min^−1^ for the dispersed and continuous phases, respectively. The droplets produced were stored in the droplet wells, after which the charged fluorosurfactants (KRYTOX, M4SURF) were introduced at a constant flow rate of 2 μL min^−1^. The fluorescent images were taken every 2 s for 10 min to observe the temporal changes inside the droplets.

### MCF-7_eGFP_ cell culture and encapsulation

Human MCF-7 epithelial breast cancer cells expressing the Epidermal Growth Factor Receptor (EGFR)^54^ genetically fused to enhanced green fluorescent protein (eGFP-EGFR) were cultured under standard conditions (95% humidity and 5% CO_2_, 37 °C) in EMEM medium (Gibco) with 10% FBS, 1% penicillin/streptomycin and G418 (1%) in a culture flask. The cells were split every 2 or 3 days at 80% confluency. For encapsulation of MCF-7_eGFP_ cells, the cells were prepared at a concentration of 1.5 × 10^6^–2 × 10^6^ cells per mL in fresh EMEM medium. For the encapsulation of cells in SiNP-DNA/KRYTOX droplets, the cells were premixed with SiNP-DNA (4 mg mL^−1^) just before the encapsulation and were used as the dispersed phase. KRYTOX (10 wt%) and M4SURF (10 wt%) dissolved in HFE 7500 were used as a continuous phase. For both the above experiments, PDMS chips with a flow-focusing junction and droplet wells (see Fig. S3†) were used with flow rates of 0.5 μL min^−1^ and 10 μL min^−1^ for the dispersed phase and continuous phase, respectively.

## Conclusions

In summary, we have demonstrated for the first time a novel electrostatic assembly of DNA-functionalized silica nanoparticles (DNA-SiNP) and charged fluorosurfactants at the interface of W/FO droplets. Using two differently charged fluorosurfactants, the commercially available negatively charged KRYTOX and the first-time synthesized positively charged M4SURF, we found practical ways to recruit and assemble DNA-SiNP into thin films at the otherwise inert inner droplet interface of W/FO water droplets. The multi-component reaction (MCR) strategy used for the synthesis of M4SURF for the first time offers a wide range of possibilities for the synthesis of fluorosurfactants, since a large variety of surfactants with almost arbitrary head groups can be synthesized by MCR. For example, additional (bio)functional groups, such as biotin or specific ligands for cell surface receptors, could thus also be made available directly by the fluorosurfactant at the droplet interface for interaction with the encapsulated content.

Our work also contributes to the mechanistic understanding of interfacial phenomena in W/FO emulsions. By using the microfluidic platform with fixed droplet positions in combination with real-time fluorescence monitoring and automated image analysis of the droplets, we were able to quantify the segregation kinetics of DNA-SiNP in the W/FO droplets with high throughput. Thus, we demonstrated that both the variation of the components in the oil phase (charge and structure of the fluorosurfactant) and in the aqueous phase (ionic conditions) affect the segregation and assembly rate of the nanoparticles in the aqueous phase. Since this methodological approach allows quantitative assessment of nanoparticle self-assembly, it can be advantageously used for future development of other complex biofunctional interfaces in microfluidic droplets.

As a proof-of-concept, we also demonstrated that the formed microfluidic droplets can be used as hollow sphere 3D microreactors for encapsulating single adherent MCF-7_eGFP_ cells. Although storage of the droplets for extended periods of time to allow cell growth and division remains an unresolved challenge to date, the SiNP-modified W/FO droplets may provide an ideal platform for further studies because, in principle, they allow long-lasting cultivation of cells due to the gas permeability of the fluorocarbon oils, and because the self-assembled SiNP-DNA layer can be tuned in many ways to serve as a tailored cell culture substrate for adherent cells. We anticipate that the functionalization of the W/FO droplet interface described above will open the door to a broader applicability of nanoparticle building blocks to modify the inner surface of microfluidic droplets. By using well-established bioconjugation strategies for nanoparticles, especially those based on DNA hybridization, a wide variety of different biomolecules such as proteins, ligands (*e.g.*, the epidermal growth factor EGF), aptamers, or RNA could thus be bound to the SiNP in order to use SiNP-based hollow sphere microreactors for cell culture applications of adherent cells.

## Author contributions

C. M. N. and S. S. designed the project and conceptualized the presented idea. B. H. and H. M. designed the synthetic strategy for M4SURF and performed synthesis and characterization of M4SURF. J. S. and R. M. provided a MATLAB pipeline for kinetic evaluation of droplets. S. S. performed microfluidic experiments, kinetic evaluation, interfacial tension measurements and cell encapsulation experiments. C. M. D., T. S., and H. M. contributed to experimental discussions, data analysis and manuscript preparation. S. S., C. M. D. and C. M. N. wrote the manuscript. All authors discussed and contributed to the final manuscript.

## Conflicts of interest

There are no conflicts to declare.

## Supplementary Material

NA-005-D3NA00124E-s001
