# Supplementary material for: Charge controlled interactions between DNA-modified silica nanoparticles and fluorosurfactants in microfluidic water-in-oil droplets
Source: Nanoscale Adv. 2023 Jul 5;5(15):3914–23. doi: 10.1039/d3na00124e (PMC10367961; doi:10.1039/d3na00124e)
Supplement: NA-005-D3NA00124E-s001 [file NA-005-D3NA00124E-s001.pdf]

Electronic Supplementary Material (ESI) for Nanoscale Advances.
This journal is © The Royal Society of Chemistry 2023

Supporting Information

Charge-controlled interactions between DNA-modified silica nanoparticles and fluorosurfactants in microfluidic water-in-oil droplets

*Sahana Sheshachala, [a] Birgit Huber, [b] Jan Schuetzke, [c] Ralf Mikut, [c] Tim Scharnweber, [a] Carmen M. Domínguez, *[a] Hatice Mutlu *[b] and Christof M. Niemeyer *[a]*

[a] Institute for Biological Interfaces (IBG 1), Karlsruhe Institute of Technology (KIT), Hermann-von-Helmholtz-Platz 1, 76344 Eggenstein-Leopoldshafen, Germany

[b] Soft Matter Synthesis Laboratory, Karlsruhe Institute of Technology (KIT), Hermann-von-Helmholtz-Platz 1, D-76344 Eggenstein-Leopoldshafen, Germany

[c] Institute for Automation and Applied Informatics (IAI), Karlsruhe Institute of Technology (KIT), Hermann-von-Helmholtz-Platz 1, 76344 Eggenstein-Leopoldshafen, Germany

* Corresponding authors

Table of Contents

	Page
1. Supporting Figures	2
2. Supporting Tables	10
3. References	10

1. Supporting Figures

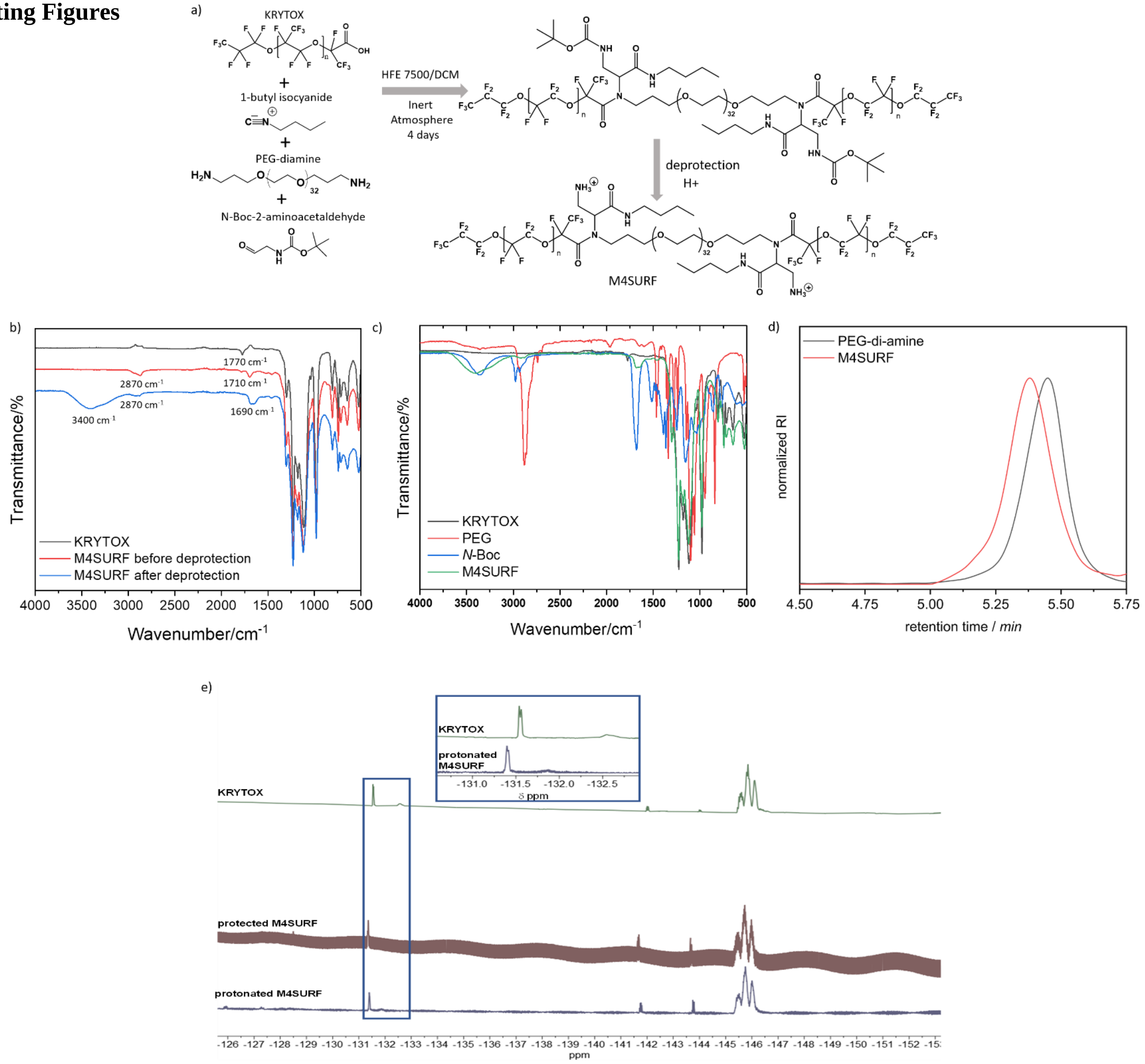

Figure S1: a) Synthesis strategy for 4 component Ugi reaction of M4SURF. b) FTIR spectra of KRYTOX, M4SURF before deprotection (red), and M4SURF after deprotection (blue). Specifically, the IR data showed that the typical KRYTOX band at 1770 cm^{-1}, which can be attributed to a stretching mode of the (C=O) bond of the PFPE-carboxylic acid of KRYTOX, had disappeared and instead a band at 1710-1690 cm^{-1} appeared, confirming the formation of a new amide bond upon the MCR. Moreover, the product revealed a characteristic band at 2870 cm^{-1} which is attributed to symmetric and asymmetric stretching modes of the PEG (C-H) groups, respectively.[1] Subsequently, the deprotection of the *N*-Boc unit in order to deliver the positively charged surfactant was verified by the appearance of the broad band at 3400 cm^{-1}, which arises due to the N–H bonded species introduced *via* protonation.[2-6] c) FTIR spectra of KRYTOX, PEG and N-boc with M4SURF product. d) Gel permeation chromatography (GPC in HFIP using PMMA standards for calibration, for details see the Experimental Section) of PEG-di-amine and M4SURF. Note that the shift in the retention time indicates the formation of M4SURF. e) ^{19}F NMR (376 MHz) spectra of the KRYTOX (green colour), protected M4SURF (brown colour) and the protonated M4SURF (violet colour) with respective zoom-in. The molecular structure of the protected M4SURF and the respective positively charged surfactant was further confirmed by ^{19}F NMR spectroscopy, as reported by Wagner[7] and Holtze et al.[8] The magnetic resonance at −132.54 ppm was attributed to the fluorine absorption vibration on the adjacent PFPE carboxyl carbon atom in KRYTOX. The sharp peak at −131.56 ppm was assigned to the –CF2 group further away from the carboxylic acid head group. Upon the MCR, the fluorine absorption vibration peak shifted from −131.56 to −131.35 ppm in the protected M4SURF. Moreover, after the deprotection step, the respective magnetic resonance shifted to −131.40 ppm, respectively. All spectra were recorded in a mixture of fluorinated benzene/C6D6 (95/5 v/v%). Unfortunately, the comparative ^{1}H NMR spectra failed to provide an evident information due to solubility issues,[8] hence it is not reported.

Statement on the efficiency and sustainability of the multicomponent Ugi reaction.

Traditionally, efficiency is encoded in the synthetic chemist's mind mostly in terms of yield, selectivity and number of steps. However, the green chemistry perspective is considerably broader and includes criteria for waste generation, use of reagents and solvents, use of hazardous chemicals, energy intensity and general safety. Hence, sustainable or green chemistry has established firm ground providing essential design criteria for the development of efficient chemical syntheses of complex, high added value molecules. While multicomponent reactions have only recently been recognized as major expansion of the synthetic chemist's toolbox into the field of polymer chemistry (since 2013), there is still little awareness of the practical value of these type of reactions for meeting many of the criteria set by the green chemistry philosophy. In other words, the inherent atom economy, efficiency, mild conditions, high convergence and concomitant step economy justify how multicomponent reactions are placed in the centre of the toolbox of sustainable synthetic methodologies. In summary, the synthesis of M4SURF is environmentally friendly as at the single-reaction level we have achieved atom efficiency (i.e., near stoichiometric use of reactants without any additives), good conversions, excellent selectivity (particularly chemo- and regioselectivity) and energy efficiency. In similar manner, we acknowledge that the green performance of multicomponent chemistry would benefit from the improvement of preparative methods for isocyanides, which is the only limitation in our concept for the synthesis of M4SURF. Still, considering that the existing surfactant systems are synthesized in a relatively lengthy synthesis, requiring some skills in the art of synthetic chemistry and necessitates the use of specialized techniques and equipment, the postulated MCR appeals to be greener also in the mentioned regard.

a)

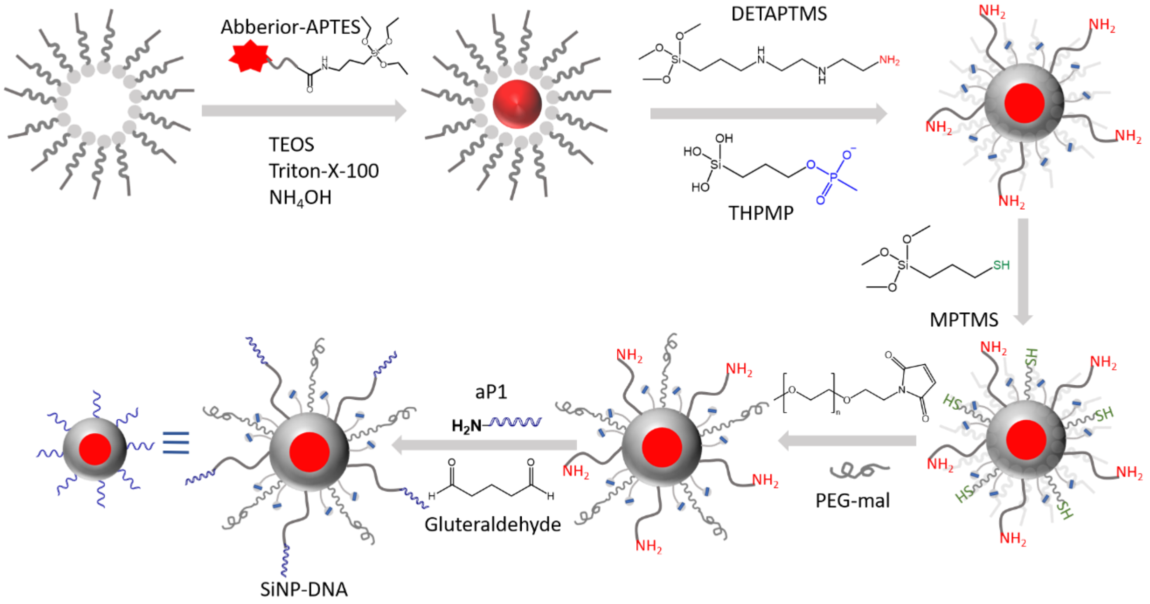

b)

SiNP	Hydrodynamic diameter (D_h)	Zeta potential (mV)	No. of DNA/molecule
SiNP-DNA	135.6 nm	-42.5	132 ± 16

c)

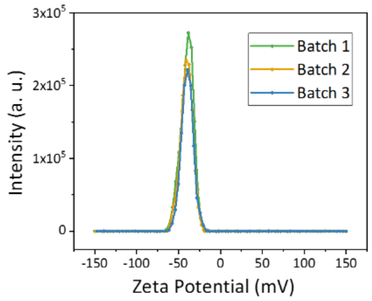

Figure S2: a) Schematic illustration of the synthesis of SiNP-DNA using the reverse micelle method. b) Physicochemical characteristics of the synthesized nanoparticles. The calculation of ssDNA per particle of SiNP was done as detailed in the previous work of Sun et al.[9] Briefly, the amount of unreacted oligonucleotides was directly quantified from the supernatant by UV/Vis after centrifugation of the reaction mixture. The number of oligonucleotides conjugated per particle was calculated by equation: $N_{ssDNA} = [(n_{added} - n_{supernatant}) \text{ x } N_A]/N_{particles}$; where N_A is 6.02×10^{23}, n_{added} is the mole of DNA added, $n_{supernatant}$ is the mole of DNA detected in the supernatant, and $N_{particles}$ is the number of particles which was calculated using the following equation: $N_{particles} = 3m/(4d \text{ x } \pi R^3)$; where m is the mass of particles, d is the particle's density of amorphous silicate (2.2 g/cm^3), π is the Archimedes' constant, and R is the radius, as determined by TEM analysis (80 nm). c) Zeta potential measured for three different batches of SiNPs.

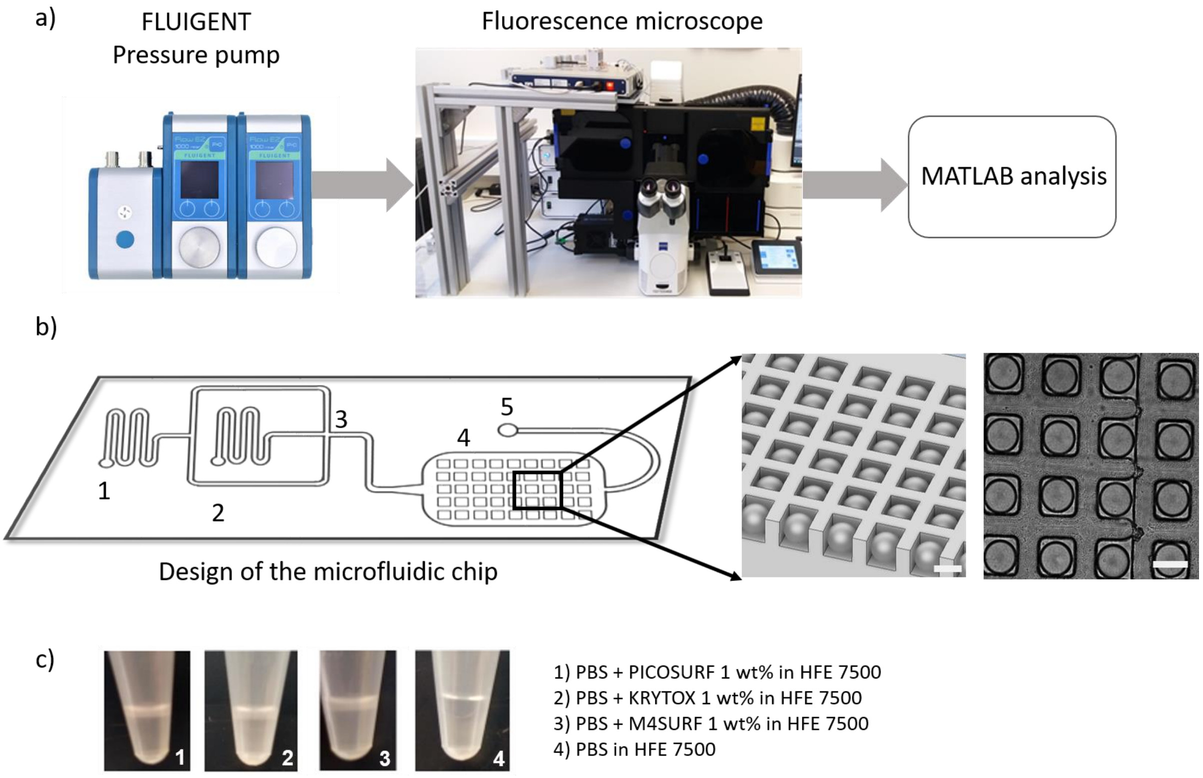

Figure S3: a) Schematic of the experimental setup and work flow for investigating the segregation of SiNP-DNA in W/FO droplets. FLUIGENT pressure pumps were used to control the flow of reagents to the microfluidic chip positioned in the focus of a high-resolution fluorescence microscope. The images were processed with a MATLAB pipeline for kinetic analysis. b) Design of the microfluidic PDMS chip consisting of two inlets (1 and 2) for the continuous and aqueous phases, respectively. The two phases meet at the flow-focusing junction (3) of cross-section 100 µm x 100 µm, where droplets are generated. The OCS chamber (4) was used as droplet pockets (OCS wells, 100 µm x 100 µm x 100 µm) to store the droplets in a fixed position. The inset also shows a schematic and brightfield image of the droplet wells. The scale bars are 100 µm. c) W/FO droplets formed with the three different surfactants used in this work could be collected using outlet (5) and stored in vials for several days at RT and 37 °C. The frothiness of the aqueous phase (top phase) indicates stable emulsion formation. Pictures were taken after storing vials at RT for 7 days.

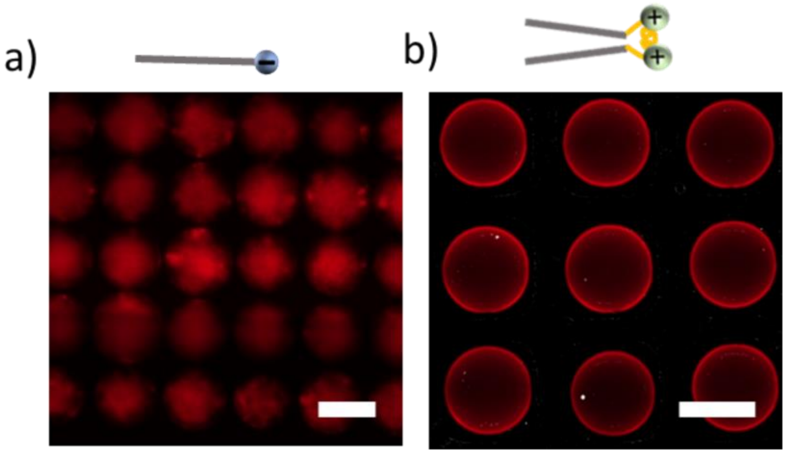

Figure S4: Fluorescence images of W/FO droplets containing SiNP-DNA in Milli-Q water in the presence of two different surfactants: (a) KRYTOX and (b) M4SURF. Scale bars are 100 µm.

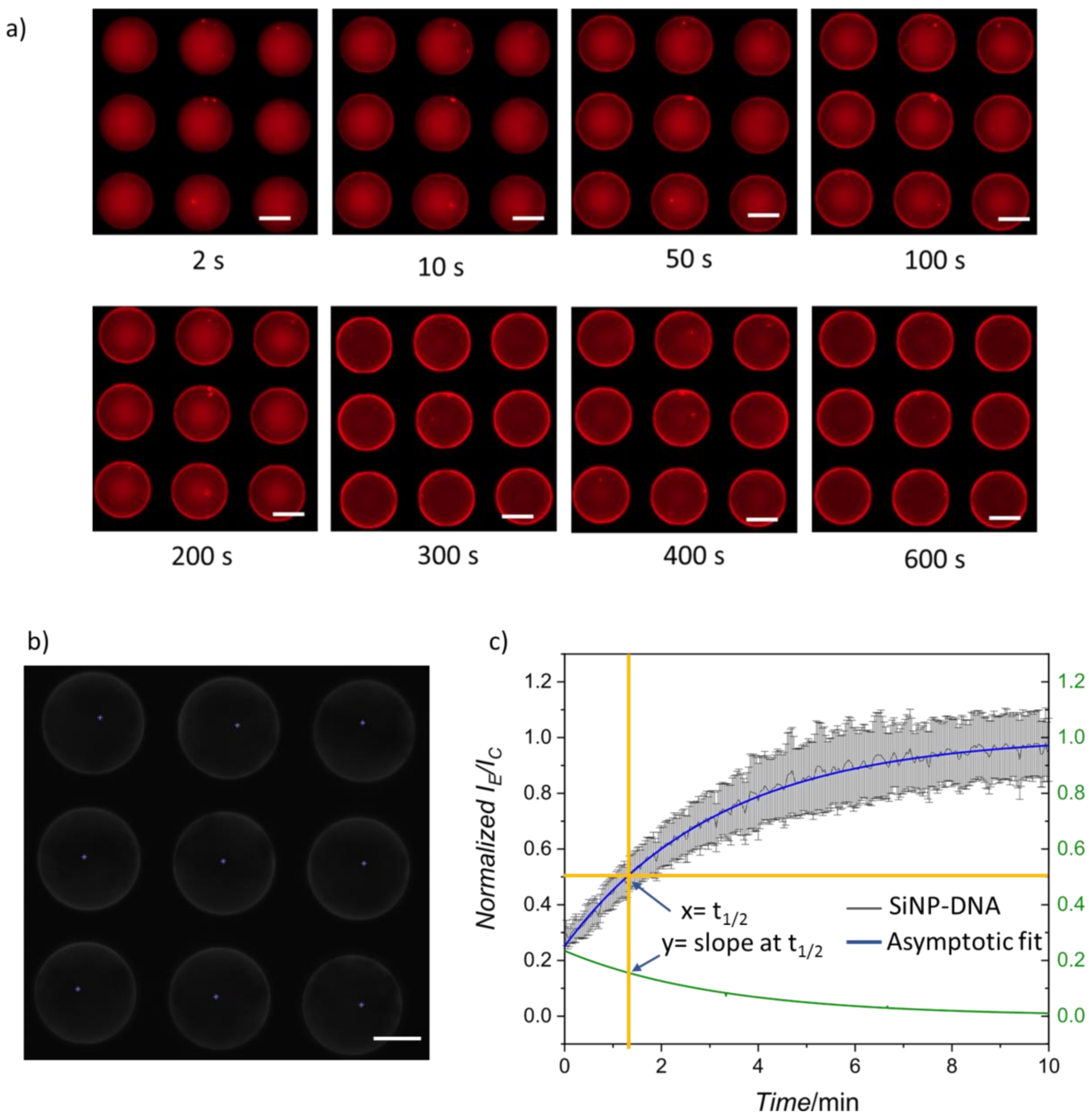

Figure S5: a) Representative fluorescent images for the temporal formation of the supramolecular assembly formed at the droplet interface after introducing either negatively charged KRYTOX or positively charged M4SURF. The scale bar is 50 µm b) Representative 8-bit grayscale droplet image recognised by the MATLAB software. The scale bar is 50 µm. c) Representative graph of time-dependent variation of intensity at edge (I_E) versus intensity at the center of the droplets (I_C) which are then converted to the normalized intensity ratios (I_E/I_C) and fitted to the asymptotic model $f(t) = a - be^{-c(t-d)}$ for the calculation of $t_{1/2}$ (intersection of yellow lines) and slope of the curve at t= $t_{1/2}$ (k_s, intersection of yellow and green line).[10]

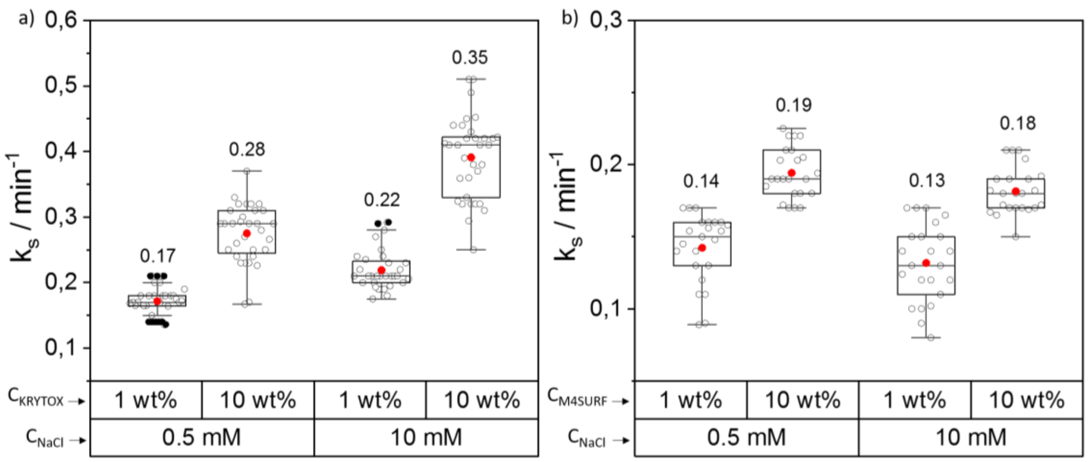

Figure S6: a) Grouped box plot of k_S values for SiNP-DNA with a varying weight percentage (1 wt% and 10 wt%) of (a) KRYTOX and (b) M4SURF in HFE oil, against concentrations of PBS containing 0.5 and 10 mM NaCl. The box plot shows median and 25-75th percentiles. Individual data points, mean and outliers are represented by hollow, red and black dots, respectively. 33 droplets were analyzed from 5 independent experiments for each condition. The corresponding mean values are also indicated above each box plot.

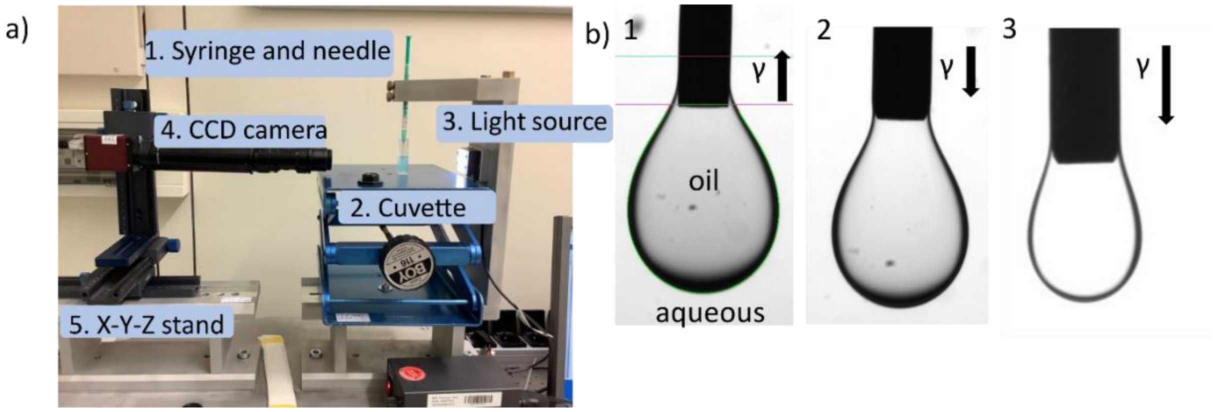

Figure S7: a) Customized setup used for pendant drop analysis to determine IFT (γ) of the SiNP-DNA/fluorosurfactants (KRYTOX and M4SURF). The HFE oil with fluorosurfactants are dispensed using (1) syringe and needle (0.8 mm) inside the aqueous solution containing SiNP-DNA placed inside the (1) cuvette. The droplets produced are illuminated using (3) light source and the droplet is captured using (4) CCD camera attached to (5) mechanical stand to adjust the position in the X-Y-Z direction. b) The γ between two immiscible phases is measured using the shape of the droplet produced using the Young-Laplace equation. An elongated droplet (2) indicates lowered γ at the oil-water interface. An extreme decrease in γ (below 4 mN/m) will also result in (3) volume reduction (smaller droplet) of the droplet formed at the oil-water interface.

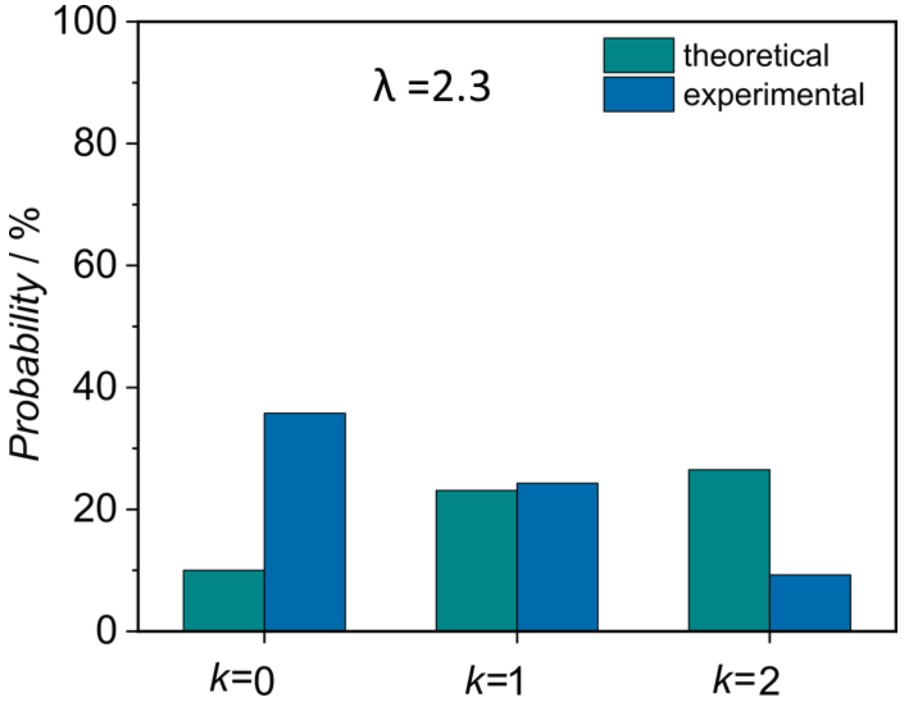

Figure S8: Statistics of MCF-7_{eGFP} cell encapsulation (experimental) compared to theoretical values obtained by Poisson distribution.[11] The number of cells encapsulated in W/FO droplets depends on the Poisson statistics due to the random distribution of cells in the cell suspension. The probability of one droplet containing k cells is dictated by

$$P(k, \lambda)=\frac{\lambda^k e^{-\lambda}}{k!},$$

where λ is the average number of cells per droplet given by

$$\lambda = C\, V_D,$$

C is the concentration of cells (2 x 10^6 cells/mL) in the dispersed phase with the units of cells/mL and V_D is the droplet volume (1150 pL) calculated using the average diameter (130 µm) measured using the MATLAB routine. The software was based on the MATLAB program and the corresponding toolbox SciXMiner.[12]

2. Supporting Tables

Table S 1: Sequence of the oligonucleotides used in this study

Oligos	Sequence	modification
aP1	TTTTTTTTTTTTTCTAACTGCTGCGCCGCCGGGAAAATACTGTACGGTTAGA	5´- (amino) C12
Cy3-aP1	AGATTGACGACGCGGCGGCCCTTTTATGACATGCCAATCT	5´- Cy3

3. References

1. Radeglia, R., Hesse, M., Meier, H. und Zeeh, B.: Spektroskopische Methoden in der organischen Chemie. VI, 478 S. 169 Abb., 86 Tab., 12 × 19 cm. Stuttgart: Georg Thieme Verlag 1979. (Thieme Taschenlehrbuch der organischen Chemie, A Theoretische und allgemeine Gebiete, Band 4) flexibles Taschenbuch, 26, 80 DM. *Journal für Praktische Chemie* **1981,** *323* (1), 175-175.
2. Kazuki, M.; Syu, H.; Tomoaki, Y.; Kazuhisa, S., Structural and Spectroscopic Characteristics of a Proton-Conductive Ionic Liquid Diethylmethylammonium Trifluoromethanesulfonate [dema][TfOH]. *Bulletin of the Chemical Society of Japan* **2010,** *83* (4), 328-334.
3. Krueger, P. J.; Smith, D. W., Amino group stretching vibrations in primary aliphatic amines. *Canadian Journal of Chemistry* **1967,** *45* (14), 1605-1610.
4. Hoque, M.; Thomas, Morgan L.; Miran, M. S.; Akiyama, M.; Marium, M.; Ueno, K.; Dokko, K.; Watanabe, M., Protic ionic liquids with primary alkylamine-derived cations: the dominance of hydrogen bonding on observed physicochemical properties. *RSC Advances* **2018,** *8* (18), 9790-9794.
5. Bellamy, L.; Williams, R., The NH stretching frequencies of primary amines. *Spectrochimica Acta* **1957,** *9* (4), 341-345.
6. Perkampus, H.-H., L. J. Bellamy: The Infrared Spectra of Complex Molecules, Vol. 1, 3. Auflage, Chapman and Hall Ltd., London 1975, 433 Seiten, 32 Abb., 22 Tabellen, Preis: £ 8.—. *Berichte der Bunsengesellschaft für physikalische Chemie* **1976,** *80* (1), 99-100.
7. Ursuegui, S.; Mosser, M.; Wagner, A., Copper-free click chemistry for microdroplet's W/O interface engineering. *RSC Advances* **2016,** *6* (97), 94942-94948.
8. Holtze, C.; Rowat, A. C.; Agresti, J. J.; Hutchison, J. B.; Angilè, F. E.; Schmitz, C. H. J.; Köster, S.; Duan, H.; Humphry, K. J.; Scanga, R. A.; Johnson, J. S.; Pisignano, D.; Weitz, D. A., Biocompatible surfactants for water-in-fluorocarbon emulsions. *Lab on a Chip* **2008,** *8* (10), 1632-1639.
9. Sun, P.; Leidner, A.; Weigel, S.; Weidler, P. G.; Heissler, S.; Scharnweber, T.; Niemeyer, C. M., Biopebble Containers: DNA-Directed Surface Assembly of Mesoporous Silica Nanoparticles for Cell Studies. *Small* **2019,** *15* (20), e1900083.
10. Sheshachala, S.; Grosche, M.; Scherr, T.; Hu, Y.; Sun, P.; Bartschat, A.; Mikut, R.; Niemeyer, C. M., Segregation of Dispersed Silica Nanoparticles in Microfluidic Water-in-Oil Droplets: A Kinetic Study. *Chemphyschem : a European journal of chemical physics and physical chemistry* **2020,** *21*, 1070 – 1078.
11. Collins, D. J.; Neild, A.; deMello, A.; Liu, A.-Q.; Ai, Y., The Poisson distribution and beyond: methods for microfluidic droplet production and single cell encapsulation. *Lab on a Chip* **2015,** *15* (17), 3439-3459.
12. Mikut, R.; Bartschat, A.; Doneit, W.; Ordiano, J. A.; Schott, B.; Stegmaier, J.; Waczowicz, S.; Reischl, M., The MATLAB Toolbox SciXMiner: User's Manual and Programmer's Guide. *ArXiv* **2017**.
